# Privacy-Preserving Outsourcing Algorithms for Multidimensional Data Encryption in Smart Grids

**DOI:** 10.3390/s22124365

**Published:** 2022-06-09

**Authors:** Feng Zhai, Ting Yang, Bing Zhao, Hao Chen

**Affiliations:** 1 School of Electrical Engineering and Automation, Tianjin University, Weijin Road No. 92, Tianjin 300072, China; zaifeng_17@tju.edu.cn; 2 China Electric Power Research Institute, State Grid, 15 Xiaoying East Road No. 15, Beijing 300072, China; zhaob@epri.sgcc.com.cn (B.Z.); chenhao2010@epri.sgcc.com.cn (H.C.)

**Keywords:** smart grid, privacy preserving, data aggregation, modular exponentiation, outsourcing computation

## Abstract

With the development of the Internet of Things, smart grids have become indispensable in our daily life and can provide people with reliable electricity generation, transmission, distribution and control. Therefore, how to design a privacy-preserving data aggregation protocol has been a research hot-spot in smart grid technology. However, these proposed protocols often contain some complex cryptographic operations, which are not suitable for resource-constrained smart meter devices. In this paper, we combine data aggregation and the outsourcing of computations to design two privacy-preserving outsourcing algorithms for the modular exponentiation operations involved in the multi-dimensional data aggregation, which can allow these smart meter devices to delegate complex computation tasks to nearby servers for computing. By utilizing our proposed outsourcing algorithms, the computational overhead of resource-constrained smart meter devices can be greatly reduced in the process of data encryption and aggregation. In addition, the proposed algorithms can protect the input’s privacy of smart meter devices and ensure that the smart meter devices can verify the correctness of results from the server with a very small computational cost. From three aspects, including security, verifiability and efficiency, we give a detailed analysis about our proposed algorithms. Finally, through carrying out some experiments, we prove that our algorithms can improve the efficiency of performing the data encryption and aggregation on the smart meter device side.

## 1. Introduction

Smart grids [1] play an important role in promoting social stability and the economic development, as they can utilize the information and communication technologies [2] to stably and efficiently generate and distribute electricity. The grid providers can formulate a more reasonable and reliable distribution strategy of electricity by the real-time collection and analysis of the situation of electric generation, transmission and demand/consumption [3]. However, the information exchange between the end user and the grid providers may face some security issues and threats [4,5,6].

The first issue is how to distinguish and authenticate the identity of a new smart meter (SM) or gateway (GW) when it wants to connect to the smart grid system [7,8]. Another issue is how to ensure the privacy and integrity of the collected data from a SM [9,10]. Integrity means to ensure that the exchanged data between the various parts of smart grid will not be modified or deleted. Privacy means to ensure that the collected data will not be leaked to the adversary. The electricity consumption data often contains the user’s confidential information. Once this confidential information is leaked or distorted, it may cause serious harm to the consumers or the grid system [11]. For example, the adversary can analyze the electricity consumption in different time periods to determine whether the owner is at home.

Recently, a number of researchers have designed many multidimensional data encryption and aggregation protocols [12,13,14] to solve the above issues. Although these protocols continually strive to improve the efficiency and enhance the protection of users’ data, these protocols often contain some complex cryptographic operations, such as modular exponentiation operation. In practical application, for the resource-constrained SM, there may be no insufficient computation resources and storage resources to carry out the modular exponentiation operation. The resource-constrained SM may take a long time to execute the designed protocol, which may not meet the requirements for real-time processing grid data. How to improve the execution efficiency of data encryption and aggregation has become an urgent problem to be solved.

Outsourcing computation [15,16] can allow the resource-constrained SM to outsource complex computation tasks to the server (may be another GW) with sufficient computation resources, which is suitable for SMs. However, there are three security challenges [17] for outsourcing computation. Trusted computing [18] may solve these challenges, but it needs corresponding hardware devices. Therefore, it has become more and more popular to design some secure outsourcing algorithms to solve these challenges. The specific challenges are described below. At first, the server is not completely trusted by the user. The outsourced data often contains the confidential data, such as the plaintext of the collected data and the user’s private key in smart grid, which cannot be disclosed to the server. The first challenge is how to protect the privacy of the user’s data in the process of outsourcing computation. Due to the various subjective factors including saving computation resources and objective factors, such as existing system bugs, attacked by hackers, the server may be curious [19,20], lazy and malicious, and thus may try to steal users’ confidential data and return random or computationally indistinguishable results to cheat users. The second challenge is to ensure that the user has the capability to verify the correctness of the returned results. The third challenge is efficiency. The time cost of the secure outsourcing algorithm on the local user side must be lower than that of solving this task by itself. Otherwise, it will be meaningless for the user to execute an outsourcing algorithm. Generally speaking, an outsourcing algorithm must be privacy-preserving, verifiable and efficient.

In this paper, we combine outsourcing computation with data encryption and aggregation to reduce the computational overhead on the SM side and improve the execution efficiency of SMs. Specifically, we explore how to outsource the modular exponentiation operation involved in these data encryption and aggregation protocols to a powerful server. In the designed outsourcing algorithms, SMs can protect the confidentiality and privacy of data by utilizing random splitting technology. In addition, a SM can verify the correctness of results from an untrusted server with a high probability and low computational overhead. We can summarize the contributions of this paper into the following aspects:

- For the different forms of modular exponentiation operation in some data encryption and aggregation protocols, we respectively propose two different outsourcing algorithms. The first outsourcing algorithm can solve modular exponentiation with a fixed base and a variable exponent. The second outsourcing algorithm can solve modular exponentiation with a variable base and exponent. Both of these two outsourcing algorithms can protect the privacy of user’s data by logic division and the Euler theorem;
- The two outsourcing algorithms ensure that a SM can verify the correctness of the returned results. In the first algorithm, an SM can detect a server’s malicious behavior with a probability of 19/20. In the second algorithm, incorrect results from a malicious server can be detected by an SM with a probability of 59/60;
- The two outsourcing algorithms only need one round of communication between the server and SM. Through systematic analysis, we can prove that the proposed algorithms satisfy all security requirements including privacy, verifiability, and efficiency. In addition, we carry out a comprehensive experiment to demonstrate that the proposed algorithms are efficient.

We organize the rest of our paper as follows: in Section 2, we review the related work. We introduce the system model and the threat model and give some formal definitions of secure outsourcing computation in Section 3. In Section 4, we briefly describe a specific data encryption and aggregation protocol and give a detailed description of the proposed outsourcing algorithms. The security and complexity analysis are given in Section 5. In Section 6, we evaluate the proposed algorithms through experiments. After that, we conclude this paper in Section 7.

## 2. Related Work

In this section, we will introduce some privacy-preserving algorithms for smart grids and review some related works about securely outsourcing modular exponentiation to a malicious server. Table 1 shows the difference between our algorithms and the previous algorithms.

### 2.1. Privacy-Preserving Algorithms for Smart Gird

Saxena et al. [23] proposed a secure and efficient mutual authentication and authorization algorithm in smart grids with an advanced metering infrastructure (AMI). The authors used an attribute-based access control to mitigate outsider and insider threats in smart grids. Their algorithm was suitable for the different user-roles scenario. However, their proposed algorithm could not consider the error detection and the fault tolerance. For secure smart grid communications, Sun et al. [24] put forward an efficient aggregation algorithm (APED) with error detection by employing a pairwise private stream aggregation method. In order to improve the APED algorithm, Shi et al. [25] proposed a diverse grouping-based algorithm (DG-APED) for data aggregation with error detection. The authors applied the differential privacy technique into the grouping-based private stream aggregation and formulated the lifetime of a SM as an exponential distribution to achieve the data aggregation and perform error detection in a malfunctioning SM. In their algorithm, a control center (CC) could only obtain the aggregated results but not the individual data. Bao et al. [26] put forward a new differentially private data aggregation algorithm with fault tolerance named DPAFT. Their algorithm could handle fault tolerance in smart metering by applying a novel key management technique and constructing an artful constraint relation. Based on an improved Boneh–Goh–Nissim cryptosystem, their algorithm could protect the privacy of the user’s data under the honest-but-curious model. Guo et al. [27] put forward a lightweight privacy-preserving data aggregation algorithm by utilizing a novel symmetric homomorphic encryption scheme. In addition, the authors also proposed an authentication agreement algorithm based on the password authenticated key exchange protocol. In order to satisfy the requirement of the fine-grained demands from CC, Li et al. [21] designed a privacy-preserving multisubset data aggregation algorithm named PPMA. Their algorithm could not only aggregate the user’s electricity consumption data of the different ranges, but also protect the privacy of user’s electricity consumption data from being disclosed to a strong adversary. Ge et al. [28] put forward a consortium blockchain-oriented approach to solve the issue of user’s privacy in energy trading in smart grids. The authors designed an account mapping technique to avoid directly exposing user’s data to attackers. Gough et al. [29] designed an innovative differential privacy-compliant algorithm to protect the data from SMs. Their algorithm ensured that the extra costs are divided among the participants by a fair, efficient and equitable manner based on the cooperative game theory.

### 2.2. Outsourcing Algorithms for Modular Exponentiation

The research on the outsourcing modular exponentiation algorithm [30,31,32,33,34,35,36] mainly focuses on two aspects: two servers and a single server. Under the two-servers model, the user can verify the correctness of the returned result by comparing the results returned from the two servers. Hohenberger et al. [30] gave a formal security definition for securely outsourcing cryptographic computation. The authors also proposed two practical and secure outsourcing algorithms for the Cramer–Shoup cryptosystem and the Schnorr signature. However, the probability that the user could detect malicious behavior from the servers was only 1/2. In order to improve efficiency and verifiability on the user side, Chen et al. [31] put forward new algorithms for the secure outsourcing of modular exponentiation. The verifiability in their algorithms was improved to 2/3. In addition, the authors also designed a secure outsourcing algorithm for simultaneous modular exponentiation. Ye et al. [32] explored how to securely outsource modular exponentiation to malicious servers by utilizing a new logical division method. The user could verify the validity of the returned results with the probability of 19/20. There are also a number of studies that concentrate on designing secure outsourcing algorithms for modular exponentiation under a single server. Wang et al. [33] firstly explored how to securely outsource modular exponentiation to a malicious server. However, the user needed to perform a time-consuming modular exponentiation operation locally once. The verifiability in their algorithm was only 1/2. Based on the Euler theorem, Ren et al. [34] presented an outsourcing algorithm for modular exponentiation under a single server. While improving efficiency, the user could check the failure with a probability of 1. However, their algorithm required the user to pre-compute some random tuples. Zhou et al. [35] put forward a secure outsourcing algorithm for modular exponentiation without pre-computation. The authors designed this new method to protect the privacy of the base, exponentiation and modular exponentiation. The user could detect malicious behavior of the server with a probability of approximately 1.

## 3. System Model and Definition

In this section, we will give a brief description about the system model and the threat model. Then, we introduce the general framework and some security requirements of the secure outsourcing computation.

### 3.1. System Model and Threat Model

**Figure 1:**
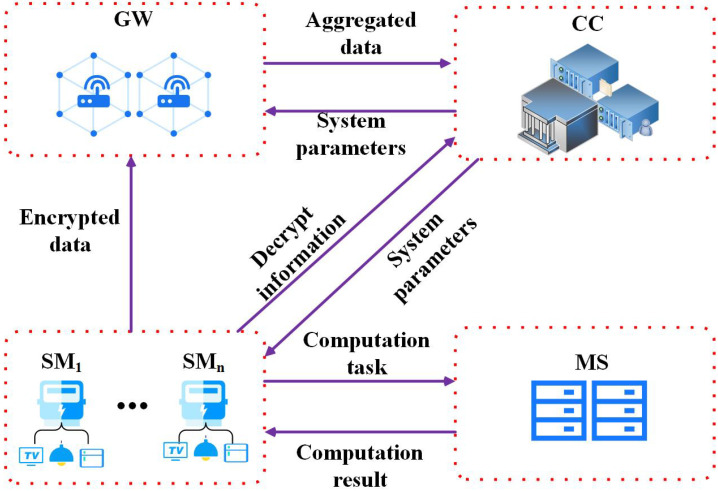
System model.

As shown in Figure 1, the system architecture of a secure outsourcing computation model in a smart grid mainly consists of four entities: smart meters (SM), gateways (GW), control centers (CC) and a malicious server (MS). A detailed description of the functions of these entities is as follows:

- SM: The main function of SMs is to continuously collect various electricity consumption information and other identity information of users, such as the user’s name and location. The SM periodically encrypts the collected data by the modular exponentiation operations and sends it to the GW;
- GW: The main function of a GW is to verify the legitimacy of the received messages and aggregate data reported by multiple SMs. Then, the GW sends the results after aggregation to the CC;
- CC: The main function of a CC is to generate system parameters. When a new device (SM or GW) is connected to the grid network, it will be authenticated by a CC. In addition, a CC can verify the legitimacy of the received messages from SMs and GWs.
- MS: The main function of a MS is to help a SM complete the corresponding complex computation tasks. The SM sends the time-consuming computation tasks to the MS, and the MS returns a correct result to the SM. The MS only communicates with the SM. There is no communication channel between the MS and the GW (or CC).

In our system architecture, we define a SM as an honest entity. The SM will carry out the corresponding operations according to protocol. However, the SM does not have sufficient computation resources and storage resources to complete complex computations. The GW and CC are honest but curious. They will execute protocol honestly, but they try to get the user’s private data during the execution of the protocol. The MS is regarded to be a malicious entity with sufficient computation resources. The MS not only wants to steal the user’s confidential data, but also returns computationally indistinguishable results to deceive the user. This threat model is called the “full-malicious” model [37] and is used in this paper. Therefore, the SM should encrypt the inputs *x* and outputs *y* of a computation task *F* before sending it to the MS and should have capability to verify the correctness of the returned results from the MS.

### 3.2. Rand Algorithm

The Rand algorithm [38] is used to generate a series of blinded pairs, shaped like $\left(c,g_{1}^{c}\right)$. The specific process of the algorithm is as follows:

$Z_{p}$ is a multiplicative group of order *M*, and $g_{1}$ is its generator. The user firstly generates *n* random integers $\alpha_{1},\alpha_{2},\ldots ,\alpha_{n}\in Z_{M}$ and computes $\beta_{j}=g_{1}^{\alpha_{j}}$. Then, the user stores $\alpha_{j}$ and $\beta_{j}$ in a table. When the user needs a blinded pair, the user will select *k* numbers from 1,2,…,n as *S*. For each j∈S, the user selects a random integer $z_{j}\in 1,2,\ldots ,h$, where h>1, and computes:

$$z\equiv \sum_{j\in S}\alpha_{j}z_{j}\left(mod\;M\right),\;\;Z\equiv \prod_{j\in S}\beta_{j}^{z_{j}}\left(mod\;p\right).$$

The user finally obtains a blinded pair (z,Z). For a *l*-bit exponent, for each invoke, the computational complexity of Rand algorithm is $O(log^{2}$l).

### 3.3. General Framework

The general framework of our proposed outsourcing algorithms contains the following sub-algorithms:

- **Pre-computation:** The SM computes some static parameters in advance to speed up the execution efficiency of algorithms;
- **Problem transformation:** At the stage of problem transformation, the SM encrypts the computation input *x* into a public value $x^{\prime }$ and stores a secret value γ locally, which can be used for decryption and verification of the results. Then, the SM sends the computation task *F* and the public value $x^{\prime }$ to the MS;
- **Server computation:** Based on the received computation task and the value $x^{\prime }$, the MS solves the transformed problem $F(x^{\prime })$ and returns the corresponding result $y^{\prime }$ to the SM;
- **Result verification:** At the stage of result verification, the SM verifies the validity the result $y^{\prime }$ based on the stored γ;
- **Result recovery:** If the result successfully passes the verification, the SM decrypts $y^{\prime }$ and obtains the real result *y*.

### 3.4. Security Requirements

We follow the security definition introduced in [30], which has been widely employed in many previous outsourcing algorithms. A cryptographic algorithm Alg can be divided into two parts: a trusted component *T* and an untrusted component *U*. *T* has access to make queries to *U*. The adversary *A* also can be divided into two parts: the adversarial environment *E* and a malicious component $U^{\prime }$ that operates in place of *U*. *E* can submit the adversarial input to Alg and $U^{\prime }$ can record all information during the execution of Alg. We consider that (T,U) is an outsource-secure implementation of Alg if (1) *T* and *U* implement Alg, i.e., $Alg=T^{U}$, and (2) a malicious $U^{\prime }$ cannot learn anything about the inputs and outputs during the execution of Alg. Furthermore, we formally define the security of the outsourcing computation for the cryptographic algorithm.

**Definition** **1** **(Algorithm with Outsource-IO).** *An algorithm*Alg*obeys outsource input/output specification if it takes in five inputs*$\left(x_{hs},x_{hp},x_{hu},x_{ap},x_{au}\right)$*and generates three outputs*$\left(y_{s},y_{p},y_{u}\right)$. *The first three inputs are generated by T and the last two inputs are adversarially submitted by E. The classification of these five inputs depends on how*$A=(E,U^{\prime })$*has knowledge about them. In particular,*

***Inputs:***

- *$x_{hs}$ is the honest and secret input, which is only known to T;*
- *$x_{hp}$ is the honest and protected input, which is known to T and E, but not to $U^{\prime }$;*
- *$x_{hu}$ is the honest and unprotected input, which is known to T, E and $U^{\prime }$;*
- *$x_{ap}$ is the adversarial and protected input, which is known to T and E, but not to $U^{\prime }$;*
- *$x_{au}$ is the adversarial and unprotected input, which is known to T, E and $U^{\prime }$.*

***Outputs:***

- *$y_{s}$ is the secret output, which is only known to T;*
- *$y_{p}$ is the protected output, which is known to T and E, but not to $U^{\prime }$;*
- *$y_{u}$ is the unprotected output, which is known to T, E and $U^{\prime }$.*

**Definition** **2** **(Outsource-Security).** *Let Alg be an algorithm with outsource-IO. A pair of algorithms (T,U) can be considered to be an outsource-secure implementation of Alg if:*

*Correctness: $T^{U}$ is a correct implementation of Alg.*

*Security: For anyone probabilistic polynomial-time adversary $A=(E,U^{\prime })$, there exists the probabilistic expected polynomial-time simulators $\left(S_{1},S_{2}\right)$ such that the following pairs of random variables are computationally indistinguishable.*

(1) **Pair One:** $EVIEW_{real}∼EVIEW_{ideal}$.

If the random variables of **pair one** are computationally indistinguishable, this means the adversarial environment *E* cannot learn any useful information.

We can formally define the view obtained by the adversarial environment *E* in the real processing process as follows:

$$\begin{array}{ccc} \; & \; & EVIEW_{real}^{i}=\{\left(IS^{i},x_{hs}^{i},x_{hp}^{i},x_{hu}^{i}\right)\leftarrow I\left(1^{k},IS^{i-1}\right);\\ \; & \; & \left(ES^{i},j^{i},x_{ap}^{i},x_{au}^{i},stop^{i}\right)\leftarrow E\left(1^{k},EVIEW_{real}^{i-1},x_{hp}^{i},x_{hu}^{i}\right);\\ \; & \; & (TS^{i},US^{i},y_{s}^{i},y_{p}^{i},y_{u}^{i})\leftarrow \\ \; & \; & T^{U^{\prime }\left(US^{i-1}\right)}\left(TS^{i-1},x_{hs}^{j^{i}},x_{hp}^{j^{i}},x_{hu}^{j^{i}},x_{ap}^{i},x_{au}^{i}\right):\\ \; & \; & (ES^{i},y_{p}^{i},y_{u}^{i})\}.\\ \; & \; & EVIEW_{real}=EVIEW_{real}^{i}\;if\;stop^{i}=TRUE. \end{array}$$

The real process proceeds in rounds. There is an honest and stateful process *I*, which the environment *E* does not have access to. In round *i*, *I* can pick the honest (secret, protected and unprotected) inputs.

Then, *E* can choose some parameter values according to its view from the last round. The value of $ES^{i}$ is a way of remembering what it does next time when it is invoked. $j^{i}$ is the index of inputs $\left(x_{hs}^{i},x_{hp}^{i},x_{au}^{i}\right)$ (*E* can only specify the index, but cannot specify their values of these inputs). *E* can choose the adversarial and protected input $x_{ap}^{i}$ and the adversarial and unprotected input $x_{au}^{i}$. $stop^{i}$ is the Boolean variable, which determines whether the round *i* is the last round in the process.

Based on the inputs $\left(TS^{i-1},x_{hs}^{j^{i}},x_{hp}^{j^{i}},x_{hu}^{j^{i}},x_{ap}^{i},x_{au}^{i}\right)$, the algorithm $T^{U^{\prime }}$ begins to run, where $TS^{i-1}$ is previously saved by *T* and generates a new state $TS^{i}$ for *T*. In addition, $T^{U^{\prime }}$ produces the secret output $y_{s}^{i}$, the protected output $y_{p}^{i}$ and the unprotected output $y_{u}^{i}$. The oracle $U^{\prime }$ is given its previously saved state $US^{i-1}$, as input, and the current state of $U^{\prime }$ is saved in the variable $US^{i}$. *E* can obtain the final view in the real process, which is just its view in the last round.

$$\begin{array}{ccc} \; & \; & EVIEW_{ideal}^{i}=\{\left(IS^{i},x_{hs}^{i},x_{hp}^{i},x_{hu}^{i}\right)\leftarrow I\left(1^{k},IS^{i-1}\right);\\ \; & \; & \left(ES^{i},j^{i},x_{ap}^{i},x_{au}^{i},stop^{i}\right)\leftarrow E\left(1^{k},EVIEW_{ideal}^{i-1},x_{hp}^{i},x_{hu}^{i}\right);\\ \; & \; & \left(AS^{i},y_{s}^{i},y_{p}^{i},y_{u}^{i}\right)\leftarrow Alg\left(AS^{i-1},x_{hs}^{j^{i}},x_{hp}^{j^{i}},x_{hu}^{j^{i}},x_{ap}^{i},x_{au}^{i}\right);\\ \; & \; & (SS^{i},US^{i},Y_{p}^{i},Y_{u}^{i},rep^{i})\leftarrow \\ \; & \; & S_{1}^{U^{\prime }\left(US^{i-1}\right)}\left(SS^{i-1},\ldots ,x_{hp}^{j^{i}},x_{hu}^{j^{i}},x_{ap}^{i},x_{au}^{i},y_{p}^{i},y_{u}^{i}\right);\\ \; & \; & \left(z_{p}^{i},z_{u}^{i}\right)=rep^{i}\left(Y_{p}^{i},Y_{u}^{i}\right)+\left(1-rep^{i}\right)\left(y_{p}^{i},y_{u}^{i}\right):\\ \; & \; & (ES^{i},z_{p}^{i},z_{u}^{i})\}.\\ \; & \; & EVIEW_{ideal}=EVIEW_{ideal}^{i}\;if\;stop^{i}=TRUE. \end{array}$$

The ideal process also proceeds in rounds. In the ideal process, there is a stateful simulator $S_{1}$, which can obtain the view when shielded from the secret input $x_{hs}^{i}$. In round *i*, based on the non-secret outputs that Alg generates, $S_{1}$ can decide to either output the values $\left(y_{p}^{i},y_{u}^{i}\right)$ generated by Alg or replace them with some other values $\left(Y_{p}^{i},Y_{u}^{i}\right)$. Whether $y_{p}^{i}$ will be replaced with $Y_{p}^{i}$ depends on the value of $rep^{i}$, which is a bit. In doing this, $S_{1}$ is allowed to query the oracle $U^{\prime }$; additionally, $U^{\prime }$ saves its state as in the real experiment.

(2) **Pair Two:** $UVIEW_{real}∼UVIEW_{ideal}$.

If the random variables of **pair two** are computationally indistinguishable, this means the malicious software $U^{\prime }$ cannot learn any useful information.

The view that the untrusted software $U^{\prime }$ can obtain in the real process is the same as that described in **pair one**. $UVIEW_{real}=US^{i}\;if\;stop^{i}=TRUE$.

$$\begin{array}{ccc} \; & \; & UVIEW_{ideal}^{i}=\{\left(IS^{i},x_{hs}^{i},x_{hp}^{i},x_{hu}^{i}\right)\leftarrow I\left(1^{k},IS^{i-1}\right);\\ \; & \; & (ES^{i},j^{i},x_{ap}^{i},x_{au}^{i},stop^{i})\leftarrow \\ \; & \; & E(1^{k},ES^{i-1},x_{hp}^{i},x_{hu}^{i},y_{p}^{i-1},y_{u}^{i-1});\\ \; & \; & \left(AS^{i},y_{s}^{i},y_{p}^{i},y_{u}^{i}\right)\leftarrow Alg\left(AS^{i-1},x_{hs}^{j^{i}},x_{hp}^{j^{i}},x_{hu}^{j^{i}},x_{ap}^{i},x_{au}^{i}\right);\\ \; & \; & \left(SS^{i},US^{i}\right)\leftarrow S_{2}^{U^{\prime }\left(US^{i-1}\right)}\left(SS^{i-1},x_{hu}^{j^{i}},x_{au}^{i}\right):\\ \; & \; & (US^{i})\}.\\ \; & \; & UVIEW_{ideal}=UVIEW_{ideal}^{i}\;if\;stop^{i}=TRUE. \end{array}$$

In the ideal process, there is a stateful simulator $S_{2}$, which can only know the unprotected input $\left(x_{hu}^{i},x_{au}^{i}\right)$ and has the access to query $U^{\prime }$. As before, $U^{\prime }$ may maintain its state.

**Definition** **3** **(α-Efficient [39]).** *A pair of algorithms $\left(T,U^{\prime }\right)$ can be considered to be an α-efficient implementation of an algorithm Alg if (1) $\left(T,U^{\prime }\right)$ is an outsource-secure implementation of Alg, and (2) for any inputs x, we suppose that the time cost for a task F to be solved locally by T is $t_{1}$ and the time cost for a task F to be solved locally by a secure outsourcing algorithm is $t_{2}$, which satisfies $\frac{t_{2}}{t_{1}}\leq \alpha$.*

**Definition** **4** **(β-Verifiable [39]).** *A pair of algorithms $\left(T,U^{\prime }\right)$ can be considered to be a β-verifiable implementation of an algorithm Alg if (1) $\left(T,U^{\prime }\right)$ is an outsource-secure implementation of Alg, and (2) for any inputs x, if there exists the malicious behavior from $U^{\prime }$, the probability that T can detect the error is not less than β.*

**Remark** **1.** *In this paper, we do not adopt a method [40] to show the security of our proposed algorithms in a real environment. We only prove that our proposed algorithms meet our proposed security definition through detailed theoretical analysis. In the future research, we will explore how to prove the security in a real environment according to the method proposed in [40].*

## 4. Algorithm

Recently, Zuo et al. [12] put forward a privacy-preserving aggregation algorithm for multidimensional data in a smart grid without a trusted authority. Their algorithm is based on the ElGamal cryptosystem, which can support distributed decryption. This algorithm needs the SM to encrypt the collected data periodically by the ElGamal encryption in the data encryption stage. We will use Zuo’s algorithm as an example to describe our algorithm.

In this section, we will briefly describe Zuo’s algorithm. Then, for the modular exponentiation operation in Zuo’s algorithm, we give a detailed description of our proposed secure outsourcing algorithms.

### 4.1. Overview of Zuo’s Algorithm

In Zuo’s algorithm, there are six parts in total. We assume that the number of SM is *n*.

1. **System Initialization**: CC firstly generates a series of system parameters $\left\{G,G_{T},g,e,H,pk_{CC},\vec{a},\vec{b},\left(R_{1},R_{2},\ldots ,R_{k},E\right)\right\}$, where *G* and $G_{T}$ are the multiplicative cyclic group of a large secure prime *p*, *g* is a generator of *G*, *e* is a bilinear map: $G\times G\to G_{T}$, *H* is a one-way hash function: ${\left\{0,1\right\}}^{*}\to G$, $pk_{CC}$ is the public key of CC, $\vec{a}=\left\{a_{1},a_{2},\ldots ,a_{w}\right\}$ and $\vec{b}=\left\{b_{1},b_{2},\ldots ,b_{k}\right\}$ are the superincreasing sequences, and $\left(R_{1},R_{2},\ldots ,R_{k},E\right)$ are the *k* range values of power consumption.
2. **Registration**: All ${\mathrm{SM}}_{i}$ and GW register with CC. $SM_{i}$ chooses a random number $x_{i}\in Z_{p}$ and computes the public key $pk_{i}=g^{x_{i}}$ and the signature $\sigma_{i}=H(ID_{i}\left|\right|T_{i}{)}^{x_{i}}$, where $ID_{i}$ is the identity of the user $U_{i}$ and $T_{i}$ is the current timestamp. Then, ${\mathrm{SM}}_{i}$ sends $\left(ID_{i},T_{i},\sigma_{i},pk_{i}\right)$ to the CC. The CC verifies if $e\left(g,\sigma_{i}\right)=e(pk_{i},H(ID_{i}\left|\right|T_{i}))$ holds. Once the equation is established, it means that the registration is successful. The registration progress of the GW is similar. The GW randomly chooses a number $x_{GW}$ and computes the public key $pk_{GW}$.
3. **Generation of Common Public Key**: Each ${\mathrm{SM}}_{i}$ broadcasts its public key $pk_{i}$ and verifies the validity of other public keys from other SMs. Then, ${\mathrm{SM}}_{i}$ computes the common public key PK as follows:
  $$PK=\prod_{i=1}^{n}pk_{i}=g^{x_{1}+x_{2}+\ldots +x_{n}}.$$
4. **Encryption of User Data**: Each ${\mathrm{SM}}_{i}$ collects *w* dimensions of power consumption $\left(m_{i1},m_{i2},\ldots ,m_{iw}\right)$. $m_{i}=\left(m_{i1}+m_{i2}+\ldots +m_{iw}\right)$ is the total power consumption data of each user. If $m_{i}\in \left[R_{j},R_{j+1}\right)$, ${\mathrm{SM}}_{i}$ chooses a random number $r_{i}\in Z_{p}^{*}$ and computes the corresponding ciphertext $\left(C_{i}^{a},C_{i}^{b}\right)$ based on the common public key PK and $g^{b_{j}}$, where i=1,2,…,n and j=1,2,…,k.
  $$\begin{array}{cc} C_{i}^{a} & =g^{r_{i}},\\ C_{i}^{b} & =g^{a_{1}m_{i1}+a_{2}m_{i2}+\ldots +a_{w}m_{iw}}\cdot g^{b_{j}}\cdot PK^{r_{i}}. \end{array}$$
  ${\mathrm{SM}}_{i}$ computes its own signature as follows:
  $$\sigma_{i}=H(ID_{i}\left|\right|C_{i}^{a}\left|\right|C_{i}^{b}\left|\right|T_{i}{)}^{x_{i}}.$$
  ${\mathrm{SM}}_{i}$ sends $\left\{ID_{i},C_{i}^{a},C_{i}^{b},T_{i},\sigma_{i},pk_{i}\right\}$ to the GW.
5. **Data Aggregation**: The GW firstly checks the timestamp $T_{i}$ and verifies the validity of *n* signatures. After successful verification, the GW computes the aggregated ciphertext $\left(C^{a},C^{b}\right)=\left(\prod_{i=1}^{n}C_{i}^{a},\prod_{i=1}^{n}C_{i}^{b}\right)$ and its own signature $\sigma_{GW}$. The GW sends $\{ID_{GW},C^{a},C^{b}$,$T_{GW},$$\sigma_{GW},pk_{GW}\}$ to the CC.
6. **Decryption of aggregation Data**: The CC firstly checks the timestamp $T_{GW}$ and verifies the signature. Each ${\mathrm{SM}}_{i}$ is required to provide $D_{i}$ and signature $\sigma_{i}^{d}$ to the CC.
  $$\begin{array}{cc} D_{i} & ={\left(C^{a}\right)}^{x_{i}}={\left(\prod_{i=1}^{n}C_{i}^{a}\right)}^{x_{i}},\\ \sigma_{i}^{d} & =H(ID_{i}\left|\right|D_{i}\left|\right|T_{i}^{d}{)}^{x_{i}}. \end{array}$$
  where $T_{i}^{d}$ is the current timestamp. ${\mathrm{SM}}_{i}$ sends $\left\{ID_{i},D_{i},T_{i}^{d},\sigma_{i}^{d},pk_{i}\right\}$ to the CC. The CC recovers the aggregated data.

From the above description, we can know that there are many complex and time-consuming cryptography operations in Zuo’s algorithm, including the modular exponentiation operation and the bilinear pairing operation. For the GW and CC, there are sufficient computation resources and storage resources to carry out these complex operations. However, for an SM, as a kind of resource-constrained device, there are not enough computation resources and storage resources to deal with the time-consuming modular exponentiation operation, or it needs a lot of time to complete these operations. In Zuo’s algorithm, there are three parts involving modular exponentiation operations: registration, encryption and decryption. In the registration process, each SM only performs a modular exponentiation operation. Even if it takes a longer time, it has little effect on the efficiency of the protocol. We mainly solve the modular exponentiation operation in encryption and decryption because the SM constantly performs modular exponentiation. This situation is not suitable for the CC to process power consumption data in real time. Therefore, we design secure outsourcing algorithms to improve the efficiency of the SM’s execution of modular exponentiation.

### 4.2. Description of Outsourcing Algorithms

In Zuo’s algorithm, every time the SM sends power consumption data, the SM needs to perform the modular exponentiation operation five times. The SM needs to compute $C_{i}^{a}$, $C_{i}^{b}$, $\sigma_{i}$, $D_{i}$ and $\sigma_{i}^{d}$. When calculating $C_{i}^{a}$ and $C_{i}^{b}$, the base of modular exponentiation is fixed and secret, and the exponent is variable and secret. When calculating $\sigma_{i}$, $D_{i}$ and $\sigma_{i}^{d}$, the base and exponent of modular exponentiation are variable and secret. For these two different cases of modular exponentiation operations, we design two different secure outsourcing algorithms.

We use *u* to represent the base of modular exponentiation and *a* to represent the exponent of modular exponentiation. *p* is the modulus. The SM sends *u*, *a* and *p* to the MS, and the MS returns $u^{a}\;\mathrm{mod}\;p$ to the SM. In the process of computing $C_{i}^{a}$ and $C_{i}^{b}$, *g* is the generator of a group and PK is the common public key. *g* and PK are the fixed parameters. When designing an outsourcing algorithm for $C_{i}^{a}$ and $C_{i}^{b}$, the SM needs to protect the privacy of *u*, *a* and *p*.

In Algorithm 1, *u* and *p* are secret and fixed, and *a* is secret and variable. Because *u* is fixed, the SM can compute two blinding pairs, $u^{x_{1}}$ and $u^{x_{2}}$, in advance. In order to keep the information of *a* private from the MS, the SM can use $x_{1}$ and $x_{2}$ to blind the exponent *a*. Based on the discrete logarithm problem, the MS cannot obtain the base *u* from $u^{x_{1}}$ and $u^{x_{2}}$. Based on the big integer factorization problem, the MS cannot obtain the base modular *p* from sp. In order to ensure the verifiability of the returned results, the SM needs to randomly choose a number *r*. The SM not only needs to send the blinding original computation $u^{a}$ to the MS, but also needs to send the blinding-related computation $u^{ra}$ to the MS. When it is necessary to verify the correctness of the result returned from the MS, the SM only needs to verify whether the equation ${\left(u^{a}\right)}^{r}=u^{ra}$ holds. When the verification is successful, the SM can use the locally stored *p* to recovery the real result $u^{a}$.

Now, we utilize an example to illustrate our proposed Algorithm  1.

**Example** **1.** *The SM wants to compute $u^{a}\;\mathrm{mod}\;p$, where p=11 is a prime, u=2 and a=2. The SM can compute as follows:*

*1.*  *Pre-computation: The SM chooses $x_{1}=3$ and $x_{2}=7$. Then, The SM computes $u^{x_{1}}=8$ and $u^{x_{2}}=7$.*
*2.*  *Problem transformation: The SM chooses r=2 and s=3. Then, the SM computes ra=4 and sp=33. The SM computes $t_{1}=4$ and $t_{2}=2$.*
*3.*  *Server computation: The MS computes $c_{1}=4$ and $c_{2}=16$.*
*4.*  *Result verification: The SM can verify the following equation:*
$$4^{2}\;\mathrm{mod}\;11=16\;\mathrm{mod}\;11.$$
*5.*  *Result recovery: The SM can recover the real result $u^{a}=4\;\mathrm{mod}\;11=4$.*

In process of computing $\sigma_{i}$, $D_{i}$ and $\sigma_{i}^{d}$, the exponent $x_{i}$ is the user’s private key, and the base is the message summary H() or the aggregated ciphertext $C^{a}$, which cannot be disclosed to MS. Once leaked, it will have a serious impact on the security of the entire algorithm. Therefore, when designing an outsourcing algorithm for $\sigma_{i}$, $D_{i}$ and $\sigma_{i}^{d}$, the SM needs to protect the privacy of the base and the exponent, that is, the privacy of *u* and *a*.

Before introducing Algorithm 2, we firstly introduce a theorem. Let *N* be a positive integer, and let *u* be an integer which satisfies gcd(u,N)=1; then we have $u^{\phi (N)}\equiv 1\;\mathrm{mod}\;N$.

**Theorem** **1.** *For $N=p_{1}p_{2}\ldots p_{m}$, where $p_{1},p_{2},\ldots ,p_{m}$ are distinct prime numbers, we have*

$$u^{a+\gamma \phi (N)}=u^{a}\left(\;\mathrm{mod}\;N\right)$$

*where* ϕ(·) *is the Euler’s function and γ is a random integer.*

**Algorithm 1** Secure Outsourcing of Modular Exponentiation with Public Base and Secret Exponent (Fixed Base)

**Input:**
$u\in Z_{p}^{*},a\in Z_{p}^{*}$.
**Output:**
$u^{a}\;\mathrm{mod}\;p$.**1.** 
**Pre-computation:**
The SM randomly chooses $x_{1}$ and $x_{2}$ and pre-computes $u^{x_{1}}$ and $u^{x_{2}}$, where $gcd(x_{1},p-1)=1$ and $gcd(x_{2},p-1)=1$.**2.** 
**Problem transformation:**
The SM randomly chooses a number *r* from the set [2,11] and computes ra.The SM randomly chooses a larger prime *s* and computes sp.The SM computes $t_{1}$ and $t_{2}$ as:
$$\begin{array}{c} t_{1}=a/x_{1}\;\mathrm{mod}\;p-1,\;t_{2}=ra/x_{2}\;\mathrm{mod}\;p-1. \end{array}$$The SM sends sp, $u^{x_{1}}$, $u^{x_{2}}$, $t_{1}$ and $t_{2}$ to MS.**3.** 
**Server computation:**
The MS computes $c_{1}$ and $c_{2}$ as:
$$\begin{array}{c} c_{1}={\left(u^{x_{1}}\right)}^{t_{1}}\;\mathrm{mod}\;sp,\;c_{2}={\left(u^{x_{2}}\right)}^{t_{2}}\;\mathrm{mod}\;sp. \end{array}$$The MS returns $c_{1}$ and $c_{2}$ to the user.**4.** 
**Result verification:**
The SM verifies the following equation:
$${\left(c_{1}\right)}^{r}\;\mathrm{mod}\;p=c_{2}\;\mathrm{mod}\;p.$$**5.** 
**Result recovery:**
The SM can recover the real result as:
$$u^{a}=c_{1}\;\mathrm{mod}\;p.$$


In Algorithm 2, N=p and ϕ(N)=p−1. *u* and *a* are secret and variable. In order to keep the information of *u* private from the MS, the SM firstly runs the Rand algorithm to generate four blinding pairs and compute two blinding pairs to blind the base *u*. The original base *u* can be transformed into $ug_{1}^{x_{1}}$. Because the MS does not know any knowledge about $g_{1}^{x_{1}}$, the MS cannot obtain the base *u* from $ug_{1}^{x_{1}}$. By utilizing ***Theorem 1***, the SM can protect the information of *a* from being disclosed to the MS.The original exponent *a* can be transformed into $t_{3}=a+k_{1}\left(p-1\right)$, where $k_{1}$ is a random integer. Similar to Algorithm  1, the SM chooses a random integer *r* and compares the two returned results to ensure the verifiability of the results. When the verification is successful, the SM can use the locally stored $g_{1}^{x_{3}}$ and *p* to recover the real result $u^{a}$.

Now, we utilize an example to illustrate our proposed Algorithm  2.

**Example** **2.** *The SM wants to compute $u^{a}\;\mathrm{mod}\;p$, where p=11 is a prime, $g_{1}=2$, u=3 and a=2. The SM can compute as follows:*

*1.*  *Pre-computation: The SM chooses $x_{1}=1$, $x_{2}=3$, $x_{3}=2$, $x_{4}=4$, $x_{5}=7$, $x_{6}=5$. Then, SM computes $g_{1}^{x_{1}}=2$, $g_{1}^{x_{2}}=8$, $g_{1}^{x_{3}}=4$, $g_{1}^{x_{4}}=5$, $g_{1}^{x_{5}}=7$, $g_{1}^{x_{6}}=10$.*
*2.*  *Problem transformation: The SM chooses r=2, s=3$k_{1}=4$ and $k_{2}=5$. Then, the SM computes ra=4 and sp=33. The SM computes $t_{1}=2$, $t_{2}=7$, $t_{3}=42$ and $t_{4}=54$.*
*3.*  *Server computation: The MS computes $c_{1}=3$, $c_{2}=31$, $c_{3}=3$ and $c_{4}=6$.*
*4.*  *Result verification: The SM can verify the following equation:*
$$372^{2}\;\mathrm{mod}\;11=180\;\mathrm{mod}\;11.$$
*5.*  *Result recovery: The SM can recover the real result $u^{a}=372\;\mathrm{mod}\;11=9$.*

**Algorithm 2** Secure Outsourcing of Modular Exponentiation with Secret Base and Exponent (Variable Base)

**Input:**
$u\in Z_{p}^{*},a\in Z_{p}^{*}$.
**Output:**
$u^{a}\;\mathrm{mod}\;p$.**1.** 
**Pre-computation:**
The SM runs the Rand algorithm to generate four blinding pairs $\left(x_{1},g_{1}^{x_{1}}\right)$, $\left(x_{3},g_{1}^{x_{3}}\right)$, $\left(x_{4},g_{1}^{x_{4}}\right)$, $\left(x_{6},g_{1}^{x_{6}}\right)$ and randomly chooses $x_{2}$ and $x_{5}$ and computes $\left(x_{2},g_{1}^{x_{2}}\right)$ $\left(x_{5},g_{1}^{x_{5}}\right)$, where $gcd(x_{2},p-1)=1$ and $gcd(x_{5},p-1)=1$.**2.** 
**Problem transformation:**
The SM randomly chooses two numbers $k_{1}$ and $k_{2}$, and chooses a random number *r* from [2,11]. The SM computes ra.The SM randomly chooses a larger prime *s* and computes sp.The SM computes $t_{1}$ and $t_{2}$ as:
$$\begin{array}{c} t_{1}=\left(-x_{3}-x_{1}a\right)/x_{2}\;\mathrm{mod}\;p-1,\;t_{2}=\left(-x_{6}-x_{4}ra\right)/x_{5}\;\mathrm{mod}\;p-1. \end{array}$$The SM computes $t_{3}$ and $t_{4}$ as:
$$\begin{array}{c} t_{3}=a+k_{1}\left(p-1\right),\;t_{4}=ra+k_{2}\left(p-1\right). \end{array}$$The SM sends $\left(u\cdot g_{1}^{x_{1}},t_{3}\right)$, $\left(g_{1}^{x_{2}},t_{1}\right)$, $\left(u\cdot g_{1}^{x_{4}},t_{4}\right)$, $\left(g_{1}^{x_{5}},t_{2}\right)$ and sp to the MS.**3.** 
**Server computation:**
The MS computes $c_{1},c_{2},c_{3}$ and $c_{4}$ as:
$$\begin{array}{cc} c_{1} & ={\left(u\cdot g_{1}^{x_{1}}\right)}^{t_{3}}\;\mathrm{mod}\;sp,\\ c_{2} & ={\left(g_{1}^{x_{2}}\right)}^{t_{1}}\;\mathrm{mod}\;sp,\\ c_{3} & ={\left(u\cdot g_{1}^{x_{4}}\right)}^{t_{4}}\;\mathrm{mod}\;sp,\\ c_{4} & ={\left(g_{1}^{x_{5}}\right)}^{t_{2}}\;\mathrm{mod}\;sp. \end{array}$$The MS sends $c_{1},c_{2},c_{3}$ and $c_{4}$ to SM.**4.** 
**Result verification:**
The SM verifies the following equation:
$${\left(c_{1}c_{2}g_{1}^{x_{3}}\right)}^{r}\;\mathrm{mod}\;p=c_{3}c_{4}g_{1}^{x_{6}}\;\mathrm{mod}\;p.$$**5.** 
**Result recovery:**
The SM can recover the real result as:
$$u^{a}=c_{1}c_{2}g_{1}^{x_{3}}\;\mathrm{mod}\;p.$$


## 5. Security and Complexity Analysis

In this section, we firstly analyze the correctness and the security of the proposed algorithms. Then, we give a detailed description about the verifiability of the returned results. Finally, we analyze the computational complexity of the proposed algorithms.

### 5.1. Security Analysis

**Theorem** **2.** *In the malicious model, the algorithm (T,U) is an outsource-secure implementation of Algorithms 1 and 2, where the input (u,a,p) may be honest, secret; or honest, protected; or adversarial, protected.*

**Proof.**  The correctness of these two algorithms is obvious and straightforward. We mainly focus on security. $A=(E,U^{\prime })$ is a PPT adversary that interacts with a PPT algorithm *T* in the malicious model. We need to prove that **pair one** and **pair two** are computationally indistinguishable. □

**Pair One**$EVIEW_{real}∼EVIEW_{ideal}$: If the input (u,a,p) is anything other than honest and secret, the same way that the simulator $S_{1}$ behaves as in the real execution. Under an honest and secret input (u,a,p), $S_{1}$ will behave as follows: once the information is received in the *i*th round, $S_{1}$ neglects it and correspondingly makes two random queries of the form $\left(\alpha_{i},\beta_{i},\theta_{i}\right)$ to $U^{\prime }$. $S_{1}$ randomly tests one output from the program (i.e., $\beta_{i}^{\alpha_{i}}\;\mathrm{mod}\;\theta_{i})$. Once it detects an error, $S_{1}$ saves the states of itself and $U^{\prime }$, and outputs $Y_{p}^{i}=“error”,Y_{u}^{i}=\emptyset ,rep^{i}=1$. If no error is detected, $S_{1}$ outputs $Y_{p}^{i}=\emptyset ,Y_{u}^{i}=\emptyset ,rep^{i}=0$; otherwise, $S_{1}$ randomly chooses an element *R* and outputs $Y_{p}^{i}=R,Y_{u}^{i}=\emptyset ,rep^{i}=0$. In either case, $S_{1}$ also stores the appropriate states. In the real and ideal experiment, the input distributions to $U^{\prime }$ are computationally indistinguishable. The inputs in the ideal experiment are chosen uniformly at random. Each part of all two queries that *T* makes is re-randomized and computationally indistinguishable. If $U^{\prime }$ behaves honestly in the *i*th round, we have $EVIEW_{real}^{i}∼EVIEW_{ideal}^{i}$. If $U^{\prime }$ behaves dishonestly in the *i*th round, both *T* and $S_{1}$ detect malicious behavior with a high probability and output “*error*”. In the real experiment, the two outputs generated by $U^{\prime }$ are blinded by a random value. Therefore, we have $EVIEW_{real}∼EVIEW_{ideal}$. In summary, we can get $EVIEW_{real}∼EVIEW_{ideal}$ no matter whether $U^{\prime }$ is honest or malicious.

**Pair Two**$UVIEW_{real}∼UVIEW_{ideal}$: The way that the simulator $S_{2}$ behaves is as follows: Once the information is received in the *i*th round, $S_{2}$ neglects it and correspondingly makes two random queries of the form $\left(\alpha_{i},\beta_{i},\theta_{i}\right)$ to $U^{\prime }$. Then, $S_{2}$ saves the states of itself and $U^{\prime }$. These real and ideal experiments can be easily distinguished by *E* because the outputs in the ideal experiment are never corrupted. Because there is no channel between *E* and $U^{\prime }$, *E* cannot transmit any messages to $U^{\prime }$. In the *i*th round of the real experiment, *T* re-randomizes its inputs to $U^{\prime }$. $S_{2}$ generates the random and independent queries to $U^{\prime }$ in the ideal experiment. We can know $UVIEW_{real}^{i}∼UVIEW_{ideal}^{i}$. In summary, we can get $UVIEW_{real}∼UVIEW_{ideal}$.

### 5.2. Verifiability Analysis

**Theorem** **3.** *In the malicious model, the proposed Algorithm 1 is a 19/20-verifiable secure outsourcing algorithm and the proposed Algorithm 2 is a 59/60-verifiable secure outsourcing algorithm.*

**Proof.**  At first, we prove that Algorithm 1 is a 19/20-verifiable secure outsourcing algorithm. If the MS wants to cheat the SM, the MS may make some guesses about the value $c_{1},c_{2}$ and *r*. In order to verify the correctness of the results, the SM needs to check the equation:

$${\left(c_{1}\right)}^{r}\;\mathrm{mod}\;p=c_{2}\;\mathrm{mod}\;p.$$

In order to cheat the SM, the MS needs to randomly choose a integer *C* and constructs the following equation:

$${\left(Cc_{1}\right)}^{r}\;\mathrm{mod}\;p=C^{r}c_{2}\;\mathrm{mod}\;p.$$

In order to make the above equation hold, the MS needs to guess the value of *r* and distinguish $c_{1}$ from $c_{1}$ and $c_{2}$. Because the random number *r* is chosen from the set [2,11], the probability that the MS can accurately guess the value of *r* is 1/10. The probability that the MS can accurately distinguish $c_{1}$ from $c_{1}$ and $c_{2}$ is 1/2. Therefore, the probability that the MS can cheat the SM is 1/20. Algorithm 1 is a 19/20 (0.95)-verifiable secure outsourcing algorithm. □

Then, we prove that Algorithm 2 is a 59/60-verifiable secure outsourcing algorithm. Similar to Algorithm 1, in order to cheat the SM, the MS needs to randomly choose a integer *C* and construct the following equation:

$${\left(Cc_{1}c_{2}g_{1}^{x_{3}}\right)}^{r}\;\mathrm{mod}\;p=C^{r}c_{3}c_{4}g_{1}^{x_{6}}\;\mathrm{mod}\;p.$$

On the one hand, the MS needs to guess the value of *r*. On the other hand, the MS needs to distinguish $c_{1}$ and $c_{2}$ from $c_{1}$, $c_{2}$, $c_{3}$ and $c_{4}$ and separately compute $Cc_{1}c_{2}$ and $C^{r}c_{3}c_{4}$. The probability that the MS can accurately guess the value of *r* is 1/10. The probability that the MS can accurately distinguish $c_{1}$ and $c_{2}$ from $c_{1}$, $c_{2}$, $c_{3}$ and $c_{4}$ is 1/6. Therefore, the probability that the MS can cheat the SM is 1/60. Algorithm 2 is a 59/60 (0.983)-verifiable secure outsourcing algorithm.

Then, we compare the security, verifiability and efficiency of Algorithm 2 with some previous algorithms. As shown in Table 2, there is the comparison of security, verifiability and efficiency for the user in these outsourcing algorithms. In order for all outsourced algorithms to have the same level of security, we set c=r=4 and k=l=29. All random numbers $x,t_{1}$ and $t_{2}$ are larger than $2^{64}$. From Table 2, we can find that Algorithm 2 is superior to [33,41] in both efficiency and security. Compared to [42], although Algorithm 2 does less MM, it needs to do one more MInv and one Rand. However, Algorithm 2 has higher verifiability. In addition, compared with the previous three algorithms, our algorithm has higher security that can protect the modulus of modular exponentiation.

### 5.3. Complexity Analysis

**Theorem** **4.** In the malicious model, the proposed Algorithm 1 is a ((1.5log r + 3)*MM + 2*MInv)/ 1.5l*MM-efficient secure outsourcing algorithm, and the proposed Algorithm 2 is a ((1.5log r + 15)*MM + 2*MInv)/1.5l*MM-efficient secure outsourcing algorithm.

**Proof.**  We denote MM as a once modular multiplication operation and MInv as a once modular inverse operation. For a *l*-bit exponent, the SM needs to be 1.5l times MM to compute $u^{a}\;\mathrm{mod}\;p$ by the square-and-multiply method. The bit length of u,a and *p* is *l*. □

In Algorithm 1, the process of problem transformation needs three times MM (we omit ra) and twice MInv. The process of result verification needs 1.5log *r* times MM. We omit other operations such as modular additions. Thus, the proposed Algorithm 1 is a ((1.5log *r* + 3)*MM + 2*MInv)/1.5l*MM-efficient secure outsourcing algorithm.

In Algorithm 2, the process of problem transformation needs nine times MM and twice MInv. The process of result verification and recovery needs 1.5log *r*+6 times MM. Thus, the proposed Algorithm 2 is a ((1.5log *r* + 15)*MM + 2*MInv)/1.5l*MM-efficient secure outsourcing algorithm.

## 6. Evaluation

### 6.1. Numeric Analysis

In this section, we will give an analysis of communication overhead and storage space overhead.

At first, we give an analysis of communication overhead. The bit length of sp is *L*. In Algorithm 1, the SM needs to send sp, $u^{x_{1}}$, $u^{x_{2}}$, $t_{1}$ and $t_{2}$ to the MS. The size of these parameters is 4l+L bits. The MS needs to return $c_{1}$ and $c_{2}$ to the SM. The size of these parameters is 2L bits. To sum up, the communication overhead of Algorithm 1 is 4l+3L bits. Similar to Algorithm 1, in Algorithm 2, the SM needs to send $\left(u\cdot g_{1}^{x_{1}},t_{3}\right)$, $\left(g_{1}^{x_{2}},t_{1}\right)$, $\left(u\cdot g_{1}^{x_{4}},t_{4}\right)$, $\left(g_{1}^{x_{5}},t_{2}\right)$ and sp to the MS. The size of these parameters is 8l+L bits. The MS needs to return $c_{1},c_{2},c_{3}$ and $c_{4}$ to the SM. The size of these parameters is 4L bits. To sum up, the communication overhead of Algorithm 2 is 8l+5L bits.

Then, we give a analysis of storage space overhead. The storage space that the SM requires contains two parts: an online phase and offline phase. In the offline phase of Algorithm 1, the SM needs to store the two pre-computed pairs and the parameters {u,p}, which needs 6l bits storage space. In the online phase of Algorithm 1, the SM firstly needs *l* bit of storage space to store *a*. Then, the SM needs 4l+L bits storage space during the problem transformation stage, which contains the parameters $\left\{ra,s,sp,t_{1},t_{2}\right\}$. We assume that *s* and *p* have the same bit length *l* and omit the bit length of *r*. The client needs l+2L bits storage space during the result verification and recovery, which contains the parameters $\left\{c_{1},c_{2},u^{a}\right\}$. To sum up, the storage space overhead of Algorithm 1 is 12l+3L bits.

We can analyze Algorithm 2 in a similar way. The SM needs to 13l bits storage space to store six blinding pairs and *p* in the offline phase of Algorithm 2. In the online phase of Algorithm 2, the SM firstly needs 2l bits storage space to store *a* and *u*. Then, the SM needs 8l+L bits storage space during the problem transformation stage, which contains the parameters $\left\{ra,s,sp,t_{1},t_{2},t_{3},t_{4},ug_{1}^{x_{1}},ug_{1}^{x_{4}}\right\}$. We omit the bit length of *r*, $k_{1}$ and $k_{2}$. The SM needs l+4L bits of storage space during the result verification and recovery, which contains the parameters $\left\{c_{1},c_{2},c_{3},c_{4},u^{a}\right\}$. To sum up, the storage space overhead of Algorithm 2 is 24l+5L bits.

Table 3 shows the communication overhead and storage space overhead.

### 6.2. Performance Evaluation

In this section, in order to show that our proposed algorithms are efficient, we carried out some experiment evaluations. We implemented our algorithms by using the C++ programming language with the GMP library, which is specially designed to handle some large integer operations. We used a software named Sublime Text3 to write the programs. In the program, all variables were first defined as the types defined in GMP. Then, we wrote code step-by-step according to the algorithms. We need edto define many variables to receive the intermediate data. We usee the system’s own time function to compute the time cost of the two algorithms. SM was simulated by a computer with a Linux Ubuntu 20.04.2 LTS operating system and Intel Core i5 processors (2.4 GMz and 2 G memory). MS was simulated by a computer with a Linux Ubuntu 20.04.2 LTS operating system and Intel Core i5 processors (2.6 GMz and 8 G memory). In our experiment, the bit length of *p* ranged from 256 bits to 2048 bits. As shown in Table 4, there are some simulation parameters when the bit length of *p* is 256 bits and 512 bits.

**Figure 2:**
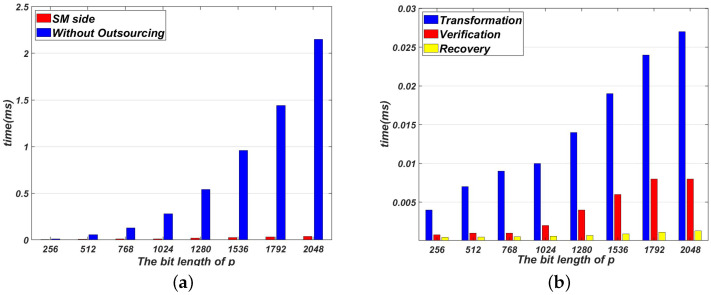
Evaluation results for Algorithm 1. (**a**) The time cost of Algorithm 1 without outsourcing on the SM side; (**b**) The time cost comparison among phases in Algorithm 1.

**Figure 3:**
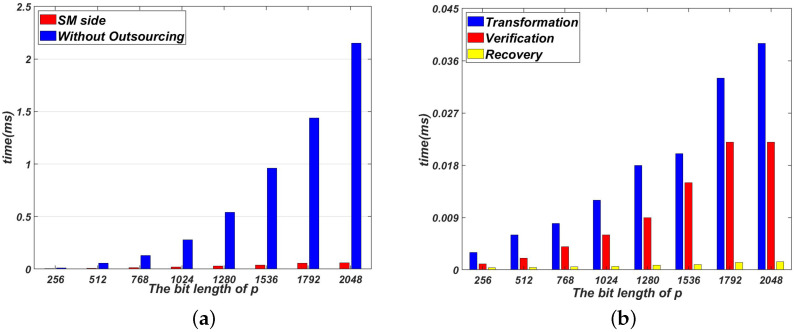
Evaluation results for Algorithm 2. (**a**) The time cost of Algorithm 2 without outsourcing on the SM side; (**b**) The time cost comparison among phases in Algorithm 2.

Figure 2a compares the time cost of not outsourcing with our proposed Algorithm 1 on the SM side. Figure 3a compares the time cost of not outsourcing with our proposed Algorithm 2 on the SM side. As shown in Figure 2a and Figure 3a, the time cost of Algorithms 1 and 2 are much smaller than that of direct computation. Note that the time cost of Algorithms 1 and 2 on the SM side dose not include the pre-computation process. The pre-computation process can be done off-line. In Algorithms 1 and 2, the time cost on the SM side concentrates on these three processes: transformation, verification and recovery.

Figure 2b and Figure 3b show the time cost of transformation, verification and recovery in Algorithms 1 and 2. As shown in Figure 2b and Figure 3b, the process of transformation needs to take more time than two other processes. This is because the process of transformation contains two modular inverse operations. Generally speaking, it takes more time to complete a modular inversion operation than a modular multiplication operation. Because only one modular operation is performed in the process of recovery, the time cost of recovery is the smallest.

**Figure 4:**
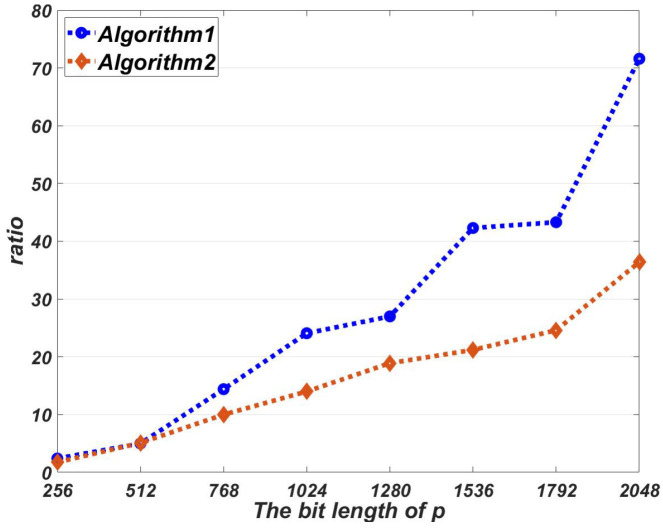
The ratio between the time cost of our proposed algorithms and direct computation.

Figure 4 shows that the ratio between the time cost of our proposed algorithms and direct computation. From Figure 4, we can see that Algorithms 1 and 2 can significantly improve the efficiency of the SM. In addition, as the bit length of *p* increases, the improvement becomes more and more significant.

## 7. Conclusions

In this paper, for the complex modular exponentiation operations involved in Zuo’s privacy-preserving data aggregation protocol, we designed two secure and efficient outsourcing algorithms for resource-constrained SMs. The proposed algorithms not only can protect SMs’ confidential data from being leaked to an untrusted server, but can also ensure the correctness of the returned results from the server. In addition, we provided an analysis of the security, verifiability and efficiency and proved that the SM can detect error with a probability of 19/20 in Algorithm 1 and with a probability of 59/60 in Algorithm 2. Finally, through experimental evaluation, we proved that our proposed algorithms are well suitable for data encryption and aggregation in smart grids. In the future, on the one hand, we will investigate more outsourcing algorithms, including bilinear pairing and scalar multiplication on elliptic curves, which are applicable to other data encryption and aggregation protocols. On the other hand, we will explore how to combine outsourcing computation with distributed computation to reduce the computational overhead on the server side.

## Figures and Tables

**Table 1:** Comparison of algorithms.

Algorithm	Data Type	Technique	Trusted Authority	Lightweight
Li [21]	One-dimensional	Paillier	Yes	No
Lu [22]	Multi-dimensional	Paillier	Yes	No
Boudia [14]	Multi-dimensional	Elliptic curve	Yes	No
Our	Multi-dimensional	Paillier	No	Yes

**Table 2:** Comparison of algorithms.

	Wang [33]	Ye [41]	Kiraz [42]	Algorithm 2
MM	12+1.5logx>108	$1.5\log r+1.5(\log t_{1}+\log t_{2})+15>210$	l+k+8logc+38=112	15+1.5logr
MInv	4	6	1	2
Rand	6	6	5	6
Verifiability	0.5	0.991	0.917	0.983
Modular Privacy	No	No	No	Yes

**Table 3:** Communication overhead and storage space overhead.

	Communication Overhead	Offline	Online	Storage Space Overhead
Algorithm 1	4l+3L	6l	6l+3L	12l+3L
Algorithm 2	8l+5L	13l	11l+5L	24l+5L

**Table 4:** Some simulation parameters.

Bit Length	*u*	*a*	*s*	*p*
256	597974957066362 8312108786874212 0805637125244242 2833798526287842 0394720897670892 71	7783106559391062 8582374740114662 1029621475861472 9700109390341782 4734058418115822 47	1086409120439892 6481456366616012 5750177785808122 7384531935993452 1304955239068542 937	8269790490761102 2215644205306412 5187816705842742 7370829104058312 0118723036268882 41
512	754387404285988 120826742806315 691191408511373 747561289674705 476743950894451 122497539359176 804470461882150 746408475691612 284449585981855 155202044474843 8435	750582098634835 192302082996469 078517856339381 182246653167329 120531958754792 548491295132292 354230821822750 054928738939141 913001392109008 976698721435666 8809	861478175873681 674678195073537 115352012738288 372116893103864 237840006599576 717793500595767 387060711833362 282500403261748 910719236579350 879254393697362 30279	783792629876206 141918443009719 853359141243678 662806276651199 939579569239026 298451825126212 286804347962362 175060120125548 509155767187344 027224581929964 0811
